# Evaluation of Pracaxi Oil as a Functional Feed Additive for Laying Japanese Quails

**DOI:** 10.1111/asj.70238

**Published:** 2026-08-31

**Authors:** Bruna de Souza Eberhart, Maria Fernanda de Castro Burbarelli, Jean Kaique Valentim, Vivian Aparecida Rios de Castilho Heiss, Felipe Cardoso Serpa, Alexander Alexandre de Almeida, Gisele Aparecida Felix, Flávia Barbieri Bacha, Claudia Andrea Lima Cardoso, Paulo Henrique Braz, Erika Rosendo de Sena Gandra, Rodrigo Garófallo Garcia, Claudia Marie Komiyama

**Affiliations:** ^1^ Faculty of Agricultural Sciences Federal University of Grande Dourados Dourados Mato Grosso do Sul Brazil; ^2^ Department of Animal Production Federal Rural University of Rio de Janeiro Seropédica Rio de Janeiro Brazil; ^3^ Greater Dourados University Center College of Veterinary Medicine Dourados Mato Grosso do Sul Brazil; ^4^ Course of Chemistry University Estadual do Mato Grosso do Sul Dourados Mato Grosso do Sul Brazil; ^5^ College of Veterinary Sciences Federal University of Fronteira Sul Chapecó Santa Catarina Brazil; ^6^ Studies Institute of Trópico Úmido Federal University of Southern and Southeastern Pará Xinguara Pará Brazil

**Keywords:** egg quality, functional oil, intestinal morphometry, nutrient metabolizability, phytogenic feed additive

## Abstract

This study evaluated the effects of dietary Pracaxi (*Pentaclethra macroloba*) oil inclusion on productive performance, egg quality, nutrient metabolizability, yolk fatty acid composition, serum biochemical parameters, organ biometry, and intestinal morphometry in laying Japanese quails. A total of 175 84‐day‐old quails were assigned to five dietary treatments containing 0%, 0.045%, 0.090%, 0.180%, and 0.360% Pracaxi oil in a completely randomized design with seven replicates of five birds over two consecutive 28‐day experimental periods. Productive performance, egg quality, serum biochemical parameters, and organ biometry were not affected by dietary treatments, although feed conversion per dozen eggs increased linearly with increasing dietary Pracaxi oil inclusion. Crude protein and mineral matter metabolizability increased linearly. Among yolk fatty acids, only α‐linolenic acid showed a quadratic response, with a maximum predicted concentration of 0.182% at a dietary Pracaxi oil inclusion level of 0.170%. Duodenal crypt depth decreased linearly, whereas the remaining intestinal morphometric variables were unaffected. Overall, dietary Pracaxi oil produced limited biological responses under the conditions evaluated, with no improvements in productive performance despite increases in crude protein and mineral matter metabolizability, changes in yolk α‐linolenic acid concentration, and reduced duodenal crypt depth. Further studies are warranted before practical recommendations can be established.

## 1 Introduction

Consumers increasingly demand poultry products free from antibiotic growth promoters due to concerns over bacterial resistance, a growing public health issue (Marshall and Levy 2011; Nhung et al. 2017). This has led researchers and producers to explore alternative strategies that maintain animal health and productivity while ensuring food safety (Ali et al. 2021). One promising option is feed additives like probiotics, prebiotics, exogenous enzymes, and phytogenic compounds, which have demonstrated potential in improving feed efficiency (Pirgozliev et al. 2019). However, the efficacy of these additives may vary according to species, physiological stage, and production system, highlighting the need for further investigation under different production conditions.

Phytogenic additives, in particular, are emerging as a sustainable alternative to traditional enhancers due to their rich bioactive content and environmentally friendly properties (Yeoman and White 2014; Abdelli et al. 2021). Plants used in traditional medicine, such as those providing carotenoids, flavonoids, and essential oils, offer a range of bioactive compounds found in seeds, leaves, flowers, and bark (Ahmad et al. 2018). Among these, *Pentaclethra macroloba* (Pracaxi) oil, native to the Amazon and surrounding regions, stands out as a promising candidate for poultry feed (Bezerra et al. 2017). Brazil's Amazon Rainforest, rich in medicinal flora, offers unique opportunities for the responsible exploitation of such natural resources (Lepsch‐Cunha et al. 2024).

Pracaxi oil is known for its antimicrobial, anti‐inflammatory, and antioxidant properties (Lamarão et al. 2023), yet its effects on poultry nutrient metabolizability, productivity, and gut health remain underexplored. Pracaxi oil is characterized by a high content of oleic acid (46.37%), behenic acid (23.01%), arachidic acid (11.98%), and linoleic acid (12.05%), which together account for more than 90% of its fatty acid composition. It also contains phenolic compounds (67.43 mg GAE g^−1^), flavonoids (30.11 mg RE g^−1^), tannins (1.03 mg ATE g^−1^), and exhibits high antioxidant capacity, as demonstrated by DPPH, FRAP, and ABTS assays (Eberhart et al. 2023). These characteristics suggest that Pracaxi oil may exert biological effects beyond its nutritional value as a lipid source, supporting its evaluation as a phytogenic feed additive.

Unlike conventional vegetable oils, which are primarily included in poultry diets as energy sources, Pracaxi oil was evaluated as a functional feed additive because of its high concentration of bioactive compounds and antioxidant constituents. Consequently, low dietary inclusion levels were adopted to investigate its biological effects independently of its contribution to dietary energy.

Phytogenic feed additives, particularly those containing plant secondary metabolites, are increasingly recognized because of their bioavailability, biological activity, and compatibility with sustainable animal production systems that enhance animal welfare and reduce environmental impact (Stevanović et al. 2018). Additionally, the sustainable use of Pracaxi oil may support local communities, non‐timber forest products, and biodiversity conservation while generating economic and cultural benefits through family farming and responsible management practices (Lepsch‐Cunha et al. 2024).

Therefore, the objective of this study was to evaluate the effects of increasing dietary Pracaxi oil inclusion on productive performance, egg quality, nutrient metabolizability, yolk fatty acid composition, serum biochemical parameters, organ biometry, and intestinal morphometry in laying Japanese quails.

## 2 Materials and Methods

### 2.1 Ethical Standards

All procedures carried out during this experiment were approved by the Ethics Committee on Animal Experimentation at the Federal University of Grande Dourados (UFGD) under protocol No. 15/2020. All procedures involving animals in this study were conducted in accordance with the guidelines of the Canadian Council on Animal Care (CCAC) to ensure the ethical and humane treatment of the animals.

### 2.2 Pracaxi Oil

For this study, Pracaxi oil was provided by a commercial company specialized in the collection and processing of Pracaxi seeds for oil extraction by cold pressing. The seeds were sourced from the Amazon Rainforest, primarily from the states of Pará and Amazonas, and processed in Belém, Pará, Brazil, by Amazon Oil.

The oil was 100% pure and natural, obtained by cold‐pressed extraction, with drying temperatures not exceeding 60°C. No preservatives, additives, or other chemical substances were added. The seeds were collected from wild populations naturally occurring in the Amazon Rainforest, and the extraction process followed sustainable harvesting practices without the use of pesticides or fertilizers (Amazon Oil 2024).

The dietary inclusion levels of Pracaxi oil used in this study were based on the findings of Eberhart et al. (2023), who characterized its fatty acid profile, antioxidant capacity, and phenolic compound composition, and evaluated its subchronic oral toxicity in *Rattus norvegicus* (Wistar strain).

### 2.3 Animals, Experimental Design, and Diets

An experiment was conducted to evaluate the effects of increasing dietary levels of Pracaxi oil in laying Japanese quails ( *Coturnix japonica* ) aged 84 days obtained from a commercial hatchery.

The experiment consisted of two 28‐day periods. The quails were housed in metal cages arranged in a pyramidal system, each providing a total area of 1250 cm^2^. The cages were equipped with gutter‐type feeders, nipple drinkers, and environmental control systems for lighting and temperature. The lighting program consisted of 16 h of light per day, supplemented with LED lamps.

A total of 175 quails were distributed in a completely randomized design consisting of five dietary treatments, seven replicates per treatment, and five birds per experimental unit.

The birds received corn‐ and soybean meal‐based diets formulated to meet or exceed the nutritional requirements for laying Japanese quails according to Rostagno et al. (2017), with or without Pracaxi oil supplementation. The experimental treatments consisted of a control diet (0% Pracaxi oil) and four dietary inclusion levels of Pracaxi oil.

The dietary treatments were as follows: 0, 0.045, 0.090, 0.180, and 0.360% Pracaxi oil, corresponding to 0, 450, 900, 1800, and 3600 mg kg^−1^, respectively (Table 1).

**TABLE 1:** Ingredient and calculated nutrient composition of the experimental diets containing increasing levels of Pracaxi oil.

Ingredients	Inclusion levels of Pracaxi oil (%)				
	0	0.045	0.090	0.180	0.360
Corn	56.29	56.29	56.29	56.29	56.29
Soybean meal	30.70	30.70	30.70	30.70	30.70
Limestone	6.95	6.95	6.95	6.95	6.95
Soybean oil	2.67	2.67	2.67	2.67	2.67
Inert	1.1	1.055	1.01	0.92	0.74
Dicalcium phosphate	1.02	1.02	1.02	1.02	1.02
DL‐methionine	0.41	0.41	0.41	0.41	0.41
Common salt	0.34	0.34	0.34	0.34	0.34
L‐Lysine	0.32	0.32	0.32	0.32	0.32
Vitamin premix^a^	0.1	0.1	0.1	0.1	0.1
Mineral premix^b^	0.1	0.1	0.1	0.1	0.1
Pracaxi oil	0	0.045	0.09	0.18	0.36
Calculated nutritional composition					
Metabolizable energy, kcal/kg	2800.00				
Crude protein, %	18.92				
Crude fiber, %	2.47				
Digestible lysine, %	1.15				
Methionine + cysteine, %	0.94				
Digestible methionine, %	0.66				
Calcium, %	2.99				
Available phosphorus, %	0.28				
Sodium, %	0.15				

^a^ Vitamin supplement/kg: vitamin A, 13,440,000 IU; vitamin D, 3,200,000 IU; vitamin E, 28,000 IU; vitamin K, 2880 mg; thiamine, 3500 mg; riboflavin, 9600 mg; pyridoxine, 5000 mg; cyanocobalamin, 19,200 μg; folic acid, 1600 mg; pantothenic acid, 25,000 mg; niacin, 67,200 mg; biotin, 80,000 μg; selenium, 600 ppm; antioxidant, 0.40 g.

^b^ Mineral supplement/kg: Mg, 150,000 ppm; Zn, 140,000 ppm; Fe, 100,000 ppm; Cu, 16,000 ppm; I, 1500 ppm.

### 2.4 Productive Performance

At the end of each experimental period, the remaining feed in each experimental unit was weighed and subtracted from the amount offered to determine feed intake. Feed intake was corrected for mortality by subtracting the estimated feed consumption of birds that died during the experimental period.

Egg production was recorded daily and expressed as a percentage of the average number of live birds during the period (hen‐day egg production) and relative to the number of birds housed at the beginning of the experiment (hen‐housed egg production). Marketable egg production was determined by excluding broken, cracked, soft‐shelled, and shell‐less eggs from total egg production.

During the last 3 days of each 28‐day experimental period, all intact eggs produced in each replicate were weighed to determine average egg weight. Egg mass was calculated by multiplying average egg weight by hen‐day egg production.

Feed conversion per dozen eggs was calculated as the ratio between feed intake (kg) and the number of dozens of eggs produced, whereas feed conversion per egg mass was calculated as feed intake (kg) divided by egg mass (kg).

Bird mortality was recorded daily, and bird viability (%) was calculated at the end of each experimental period.

### 2.5 Egg Quality

During the last 3 days of each experimental period, all intact eggs were collected, and two eggs per replicate per day were randomly selected for egg quality analyses, resulting in a total of 420 eggs analyzed throughout the experiment.

Eggs were individually weighed using an analytical balance (0.001 g precision), identified, and evaluated according to the methodology described by Bittencourt et al. (2019).

Specific gravity was determined using saline solutions with densities ranging from 1.060 to 1.100 g cm^−3^, at intervals of 0.005 g cm^−3^, according to the methodology proposed by Castelló et al. (1989).

After breaking the eggs, the yolk, albumen, and eggshell were separated. Yolk color was determined using a portable colorimeter (Minolta CR‐410), and the parameters lightness (L*), redness (a*), and yellowness (b*) were measured at three different points on the yolk surface. Yolk color was evaluated using the Roche Yolk Color Fan (scores ranging from 1 to 15).

Albumen height, yolk height, and yolk diameter were measured using a digital caliper mounted on a tripod. Yolk height was measured at the central region, whereas albumen height was measured approximately 4 cm from the yolk. All measurements were performed by the same evaluator.

The yolk was separated from the albumen and weighed individually. Albumen weight was calculated by difference, and eggshells were washed, dried in a forced‐air oven at 65°C for 72 h, and weighed. The percentages of yolk, albumen, and shell were calculated relative to total egg weight according to the methodology described by Pissinati et al. (2014).

Eggshell thickness was measured at three different points using a digital micrometer (Digimess, Brazil; 0.001 mm precision), and the average value was used for statistical analysis.

The Haugh unit (HU) was calculated according to Stadelman (1999) using the following equation:

$$\mathrm{HU}=100\;\log\;\left(H+7.57-1.7W^{0.37}\right)$$

where *H* is the albumen height (mm) and *W* is egg weight (g).

The yolk index was calculated as the ratio between yolk height and yolk diameter according to Moraleco et al. (2019).

For pH determination, yolk and albumen samples from two eggs per replicate were homogenized separately and measured using a digital pH meter (Testo 205), following the methodology described by Souza et al. (2021).

### 2.6 Nutrient Metabolizability

Following the experimental treatments described in Table 1, nutrient metabolizability was determined using the total excreta collection method with ferric oxide as an indigestible marker, according to Sakomura and Rostagno (2016). Immediately after the completion of the 56‐day performance trial, a five‐day total excreta collection period was initiated to determine nutrient metabolizability and nitrogen‐corrected apparent metabolizable energy (AMEn). The collection period began on day 56, and the first excreta collection was performed on day 57. Excreta were collected twice daily, at 8:00 a.m. and 4:00 p.m., for five consecutive days. All cages were fitted with trays specifically designed for excreta collection.

Excreta were collected, placed in plastic bags, identified by experimental unit, and stored at **−**16°C. At the end of the collection period, feed intake and total excreta production were recorded. The excreta were thawed, homogenized, and a representative sample of approximately 200 g from each experimental unit was dried in a forced‐air oven at 55°C for 72 h. After drying, the samples were equilibrated to room temperature, weighed, ground through a 1‐mm sieve using a Wiley mill, and stored at **−**20°C until laboratory analyses.

Dry matter (DM), mineral matter (MM), crude protein (CP), and ether extract (EE) were determined according to Silva and Queiroz (2002). Apparent metabolizable energy (AME), nitrogen‐corrected apparent metabolizable energy (AMEn), and metabolizability coefficients were calculated according to Matterson et al. (1965).

### 2.7 Fatty Acid Composition of Egg Yolks

At the end of the second experimental period, three eggs were collected from each experimental unit (105 eggs in total). The yolks were separated, identified, frozen, and subsequently lyophilized.

Fatty acid composition was determined by gas chromatography equipped with a flame ionization detector (FID) using a CP‐Sil 88 capillary column (60 m × 0.25 mm × 0.25 μm). Helium was used as the carrier gas at a flow rate of 0.9 mL min^−1^. The injector and detector temperatures were 250°C and 280°C, respectively, with a split ratio of 1:100.

The oven temperature program started at 175°C for 8 min, increased at 2°C min^−1^ to 180°C, remained at this temperature for 28 min, and was then increased to 250°C.

Fatty acids were identified by comparing retention times with authenticated standards, and the results were expressed as percentages of total fatty acids. All analyses were performed in triplicate according to Rodrigues et al. (2010).

### 2.8 Serum Biochemical Parameters and Organ Biometry

At the end of the second experimental period, one bird per experimental unit, with a body weight within ±5% of the respective replicate mean, was selected and subjected to a 12‐h fasting period before sampling. The selected birds were euthanized by cervical dislocation, and blood samples (3–5 mL) were immediately collected by intracardiac puncture using syringes. The samples were centrifuged to obtain serum, which was stored at −20°C until analysis.

Serum concentrations of albumin, alanine aminotransferase (ALT), aspartate aminotransferase (AST), creatinine, cholesterol, and triglycerides were determined using commercial kits according to the manufacturers' recommendations. Biochemical analyses were performed by spectrophotometry using a BioPlus 200 analyzer.

After blood collection, the birds were bled, scalded, and manually defeathered. Live weight, empty body weight, and the weights of the heart, liver, gizzard, proventriculus, intestines, reproductive tract, head, neck, and feet were recorded.

### 2.9 Intestinal Morphometry

Following organ collection, 2‐cm segments of the duodenum, jejunum, and ileum were collected, rinsed with distilled water, and fixed in 10% buffered formaldehyde, according to Tolosa et al. (2003).

Samples were dehydrated through an ascending ethanol series, cleared in xylene, embedded in paraffin, sectioned at 5 μm, and stained with hematoxylin and eosin (H&E).

Histological slides were examined using a Precision P207BI binocular microscope coupled to a BEL Engineering Eurekam 1.3 digital camera. Images were analyzed using Image‐Pro Plus software.

The following intestinal morphometric variables were measured (μm): villus height, villus width, crypt depth, and crypt diameter. The villus:crypt ratio and villus width:height ratio were subsequently calculated. Measurements were obtained from 10 well‐oriented villi per intestinal segment, according to Gava et al. (2015).

### 2.10 Statistical Analyses

The assumptions of normality and homogeneity of variances were assessed using the Shapiro–Wilk and Levene tests, respectively. After confirming these assumptions, the data were analyzed using analysis of variance (ANOVA) with the MIXED procedure of SAS (version 9.4; SAS Institute Inc., Cary, NC, USA). When significant treatment effects were detected, linear and quadratic polynomial regression analyses were performed to evaluate the responses to increasing dietary Pracaxi oil inclusion levels.

Regression models were selected based on statistical significance (***p*** < 0.05), coefficient of determination (*R* ^2^), and biological interpretability. The estimated predicted maximum inclusion levels for significant quadratic response were obtained from the fitted regression equation. Differences were considered significant at ***p*** < 0.05.

## 3 Results

The analyzed chemical composition of the experimental diets is presented in Table 2. Dry matter ranged from 86.18% to 89.72%, crude protein from 17.28% to 17.98%, ether extract from 3.02% to 3.87%, mineral matter from 13.58% to 14.59%, and gross energy from 3667% to 3703 kcal/kg. Overall, only minor variations in analyzed chemical composition were observed among the experimental diets.

**TABLE 2:** Analyzed chemical composition of the experimental diets containing increasing levels of Pracaxi oil, on an as‐fed basis.

Variables	Inclusion levels (%)				
	0	0.045	0.090	0.180	0.360
Dry matter, %	86.175	89.723	88.975	89.255	88.849
Crude protein, %	17.977	17.283	17.620	17.477	17.275
Ether extract, %	3.016	3.654	3.465	3.868	3.493
Mineral matter, %	14.213	14.591	13.855	13.626	13.577
Gross energy, kcal/kg	3667	3703	3675	3685	3698

The fatty acid profile of *Pentaclethra macroloba* oil was predominantly composed of unsaturated fatty acids (59.81%), mainly oleic acid (46.37%) and linoleic acid (12.05%), whereas saturated fatty acids accounted for 40.19%, with behenic acid (23.01%) and arachidic acid (11.98%) as the major constituents (Table 3).

**TABLE 3:** Fatty acid composition of Pracaxi (*Pentaclethra macroloba*) oil determined by gas chromatography.

Fatty acids	Composition (%)
Saturated fatty acids	
Behenic acid (C22:0)	23.01
Arachidic acid (C20:0)	11.98
Stearic acid (C18:0)	3.45
Palmitic acid (C16:0)	1.49
Lauric acid (C12:0)	0.17
Myristic acid (C14:0)	0.09
Total saturated fatty acids	40.19
Unsaturated fatty acids	
Oleic acid (C18:1)	46.37
Linoleic acid (C18:2)	12.05
Alpha‐linolenic acid (C18:3)	1.39
Total unsaturated fatty acids	59.81
Ratio of saturated to unsaturated fatty acids	0.67

Most productive performance variables were not affected by dietary Pracaxi oil supplementation (Table 4). Feed intake ranged from 28.15 to 31.54 g/bird/day, egg production from 82.62% to 88.19%, commercial egg production from 82.37% to 88.01%, viability from 94.38% to 98.93%, egg weight from 11.17 to 11.44 g, egg mass from 8.68 to 9.76 g, and feed conversion per egg mass from 3.08 to 3.61 kg/kg (*p* > 0.05). Feed conversion per dozen eggs was the only productive parameter affected, showing a positive linear response to increasing dietary Pracaxi oil inclusion (*p* = 0.0169; y = 2.577 + 1.136*x*).

**TABLE 4:** Productive performance of laying Japanese quails fed increasing dietary levels of Pracaxi oil.

Variables	Inclusion levels (%)					SEM^a^	*p*
	0	0.045	0.090	0.180	0.360		
Feed intake (g/bird/day)	28.148	28.802	31.335	29.78	31.541	0.582	0.0814
Egg production, %	83.888	82.615	88.187	87.032	85.082	1.216	0.4790
Commercial^b^	83.33	82.369	88.013	86.732	84.483	1.403	0.3730
Viability, %	98.928	98.775	97.447	97.873	94.384	0.731	0.3466
Egg mass, g	8.865	8.684	9.309	9.758	9.054	0.130	0.0740
FCR (egg mass)^c^	3.221	3.36	3.379	3.082	3.607	0.072	0.1304
FCR (dozen eggs)^d^	2.649	2.561	2.756	2.640	3.069	0.060	0.0262
Polynomial regressions							
Variable	*p*	Effect		Equations	*R* ^2^		
FCR (dozen eggs)	0.0169	Linear		*y* = 2.577 + 1.136*x*	0.822		

^a^ SEM = standard error of the mean.

^b^ Percentage of commercial eggs.

^c^ Feed conversion per egg mass.

^d^ Feed conversion per dozen eggs. Values are presented as means of seven replicates with five birds per experimental unit. Linear and quadratic effects of dietary Pracaxi oil inclusion levels were evaluated by polynomial regression. Statistical significance was declared at *p* < 0.05.

Egg quality traits were not affected by dietary treatments (Table 5). Egg weight ranged from 11.16 to 11.43 g, Haugh unit from 85.73 to 87.09, shell thickness from 0.22 to 0.24 mm, specific gravity from 1.0600 to 1.0674 g/cm^3^, and yolk pH from 6.07 to 6.16, with no significant differences among treatments (*p* > 0.05).

**TABLE 5:** Egg quality of laying Japanese quails fed increasing dietary levels of Pracaxi oil.

Variables		Inclusion levels (%)					SEM^a^	*p*
		0	0.045	0.090	0.180	0.360		
Egg weight, g		11.30	11.43	11.39	11.33	11.16	0.071	0.7471
Yolk weight, g		3.60	3.83	3.87	3.87	3.77	0.044	0.2806
Albumen weight, g		6.77	6.68	6.60	6.46	6.50	0.063	0.4229
Shell weight, g		0.92	0.91	0.91	0.92	0.87	0.007	0.0782
Albumen, %		59.92	58.43	57.86	57.37	58.08	0.384	0.2331
Yolk, %		31.88	33.58	34.10	34.39	34.06	0.384	0.1957
Shell, %		8.18	7.97	8.02	8.19	7.78	0.05	0.0526
Specific gravity, (g/cm^3^)		1.0667	1.0600	1.0662	1.0674	1.0667	0.0018	0.7694
Albumen height, mm		3.88	4.03	3.79	3.84	3.78	0.064	0.7629
Yolk height, mm		10.51	10.4	10.34	10.43	10.35	0.066	0.9393
Yolk diameter, mm		23.82	24.16	23.76	23.66	23.84	0.183	0.8190
Haugh unit		86.96	87.09	85.73	85.99	85.90	0.376	0.6808
Yolk index		0.44	0.43	0.44	0.44	0.43	0.004	0.8469
Color	L*	55.75	55.18	54.97	55.52	56.02	0.171	0.2690
	a*	−2.68	−2.63	−2.68	−2.62	−2.86	0.053	0.3809
	b*	37.24	36.65	38.31	38.52	37.99	0.244	0.0556
Shell thickness, mm		0.24	0.23	0.23	0.24	0.22	0.002	0.1354
Yolk pH		6.16	6.09	6.07	6.10	6.11	0.009	0.0679

^a^ SEM = standard error of the mean. Values are presented as means of seven replicates with five birds per experimental unit. During the last 3 days of each experimental period, two eggs per replicate per day were randomly selected for egg quality analyses (420 eggs analyzed). Linear and quadratic effects of dietary Pracaxi oil inclusion levels were evaluated by polynomial regression. Statistical significance was declared at *p* < 0.05.

Regarding nutrient metabolizability (Table 6), AMEn ranged from 2688 to 3067 kcal/kg, dry matter metabolizability from 78.34% to 83.88%, ether extract metabolizability from 87.49 to 91.38%, crude protein metabolizability from 50.43% to 77.95%, and mineral matter metabolizability from 48.28% to 50.98%. AMEn, dry matter metabolizability, and ether extract metabolizability were not affected by dietary treatments (*p* > 0.05). Crude protein metabolizability increased linearly with increasing dietary Pracaxi oil inclusion (*p* = 0.010). Likewise, mineral matter metabolizability also increased linearly with increasing dietary Pracaxi oil inclusion (*p* = 0.012).

**TABLE 6:** Nutrient metabolizability coefficients and nitrogen‐corrected apparent metabolizable energy (AMEn) of laying Japanese quails fed increasing dietary levels of Pracaxi oil.

Variables	Inclusion levels (%)					SEM^a^	*p*
	0	0.045	0.090	0.180	0.360		
AMEn^b^, Kcal/kg	2873	2688	2909	2899	3067	46.60	0.1968
Dry matter, %	81.26	78.34	83.88	80.27	83.62	1.055	0.4877
Ether extract, %	90.13	87.49	88.00	90.47	91.38	1.456	0.6362
Crude protein, %	60.16	50.43	60.42	64.85	77.95	2.905	0.0497
Mineral matter, %	48.28	48.35	49.02	50.98	50.83	0.432	0.0149
Polynomial regressions							
	*p*	Effect	Equations			*R* ^2^	
Crude protein, %	0.010	Linear	*y* = 59.35651*x* + 55.150			0.2608	
Mineral matter, %	0.012	Linear	*y* = 8.0366*x* + 48.417			0.2555	

^a^ SEM = standard error of the mean.

^b^ AMEn = nitrogen‐corrected apparent metabolizable energy. Values are presented as means of seven replicates with five birds per experimental unit. Linear and quadratic effects of dietary Pracaxi oil inclusion levels were evaluated by polynomial regression. Statistical significance was declared at *p* < 0.05.

The fatty acid composition of egg yolks was largely unaffected by dietary Pracaxi oil supplementation (Table 7). α‐Linolenic acid (C18:3 ω3) was the only fatty acid affected by dietary treatment, showing a significant quadratic response (*p* = 0.0015). Observed values ranged from 0.173% to 0.182%. The fitted regression equation predicted a maximum yolk linolenic acid concentration of 0.182% at a dietary Pracaxi oil inclusion level of 0.170%. No significant effects were observed for palmitic, palmitoleic, stearic, oleic, linoleic, arachidonic, or docosahexaenoic acids (*p* > 0.05).

**TABLE 7:** Fatty acid composition of egg yolks from laying Japanese quails fed increasing dietary levels of Pracaxi oil.

Fatty acids	Inclusion levels (%)					SEM^a^	*p*
	0	0.045	0.090	0.180	0.360		
C16:0^b^	27.836	27.927	27.944	27.873	27.945	0.014	0.0634
C16:1^c^	1.568	1.582	1.582	1.570	1.588	0.003	0.4405
C18:0^d^	9.220	9.210	9.214	9.204	9.201	0.005	0.8086
C18:1^e^	44.274	44.064	43.891	44.054	44.171	0.082	0.6864
C18:2 (ω6)^f^	13.374	13.477	13.524	13.361	13.295	0.034	0.2118
C18:3 (ω3)^g^	0.173	0.180	0.182	0.180	0.174	0.001	0.0310
C20:4 (ω6)^h^	0.191	0.191	0.188	0.195	0.192	0.001	0.6062
C22:6 (ω3)^i^	0.200	0.207	0.201	0.198	0.200	0.002	0.7201
Polynomial regressions							
	*p*	Effect	Equations		*R* ^2^		
C18:3 (ω3)	0.0015	Quadratic	*y* = −0.234*x* ^2^ + 0.080*x* + 0.175		0.2555		

^a^ SEM = standard error of the mean.

^b^ Palmitic acid.

^c^ Palmitoleic acid.

^d^ Stearic acid.

^e^ Oleic acid.

^f^ Linoleic acid (ω6).

^g^ α‐Linolenic acid (ω3).

^h^ Arachidonic acid (ω6).

^i^ Docosahexaenoic acid (ω3). Values are presented as means of seven replicates with five birds per experimental unit. Three eggs per replicate were collected at the end of the second experimental period for yolk fatty acid analysis. Linear and quadratic effects of dietary Pracaxi oil inclusion levels were evaluated by polynomial regression. Statistical significance was declared at *p* < 0.05.

Serum biochemical parameters were not influenced by dietary Pracaxi oil supplementation (Table 8). Albumin ranged from 1.72 to 1.85 g/dL, ALT from 14.20 to 33.86 U/L, AST from 129.70 to 318.20 U/L, creatinine from 66.92 to 95.74 μmol/L, cholesterol from 98.00 to 124.14 mg/dL, and triglycerides from 388.00 to 555.17 mg/dL, with no significant differences among treatments (*p* > 0.05).

**TABLE 8:** Serum biochemical parameters of laying Japanese quails fed increasing dietary levels of Pracaxi oil.

	Inclusion levels (%)					SEM^a^	*p*
	0	0.045	0.090	0.180	0.360		
Albumin, g/dL	1.830	1.722	1.847	1.757	1.797	0.163	0.9756
ALT, U/L	17.600	21.285	18.425	14.200	33.857	6.990	0.2640
AST, U/L	139.030	141.350	206.790	129.700	318.200	77.700	0.3283
Creatinine, μmol/L	95.740	68.160	66.920	94.680	83.270	12.620	0.3092
Cholesterol, mg/dL	112.330	111.860	98.000	104.500	124.140	17.560	0.8621
Triglycerides, mg/dL	555.170	416.170	388.000	416.330	455.570	84.390	0.6238

^a^ SEM = standard error of the mean. Values are presented as means of seven replicates with one bird sampled per experimental unit. Linear and quadratic effects of dietary Pracaxi oil inclusion levels were evaluated by polynomial regression. Statistical significance was declared at *p* < 0.05.

Similarly, live weight and organ biometry were not affected by dietary treatments (Table 9). Live weight ranged from 155.29 to 166.71 g, corrected live weight from 146.87 to 157.87 g, empty body weight from 97.20 to 106.56 g, liver weight from 4.18 to 4.97 g, intestinal weight from 6.56 to 8.21 g, heart weight from 1.30 to 1.43 g, reproductive tract weight from 11.53 to 12.75 g, gizzard weight from 4.01 to 4.42 g, and proventriculus weight from 0.82 to 0.93 g, with no significant differences among treatments (*p* > 0.05).

**TABLE 9:** Live weight and organ biometry of laying Japanese quails fed increasing dietary levels of Pracaxi oil.

Variable, g	Inclusion levels (%)					SEM^a^	*p*
	0	0.045	0.090	0.180	0.360		
Live weight	166.71	164.29	162.71	156.57	155.29	2.472	0.5432
Corrected LW^b^	156.21	157.87	156.62	146.87	150.93	2.542	0.6375
Empty body	103.53	101.04	106.56	97.198	97.551	1.916	0.5106
Liver	4.182	4.53	4.594	4.97	4.318	0.134	0.4110
Gut^c^	7.68	7.762	6.558	7.931	8.205	0.240	0.2412
Heart	1.334	1.39	1.382	1.431	1.304	0.027	0.6381
Reproductive^d^	11.907	12.265	11.531	12.182	12.747	0.441	0.9426
Gizzard	4.367	4.251	4.011	4.415	4.175	0.104	0.7740
Proventriculus	0.845	0.932	0.821	0.853	0.837	0.018	0.3463

^a^ SEM = standard error of the mean.

^b^ Corrected live weight.

^c^ Small and large intestine.

^d^ Complete reproductive tract. Values are presented as means of seven replicates with one bird sampled per experimental unit. Linear and quadratic effects of dietary Pracaxi oil inclusion levels were evaluated by polynomial regression. Statistical significance was declared at *p* < 0.05.

Among the intestinal morphometric variables evaluated (Table 10), only duodenal crypt depth was affected by dietary Pracaxi oil supplementation, showing a negative linear response to increasing dietary inclusion levels (*p* = 0.0008; *y* = −112.289*x* + 192.759). Villus height, villus width, crypt diameter, villus:crypt ratio, and villus width:height ratio in the duodenum, jejunum, and ileum were not affected by dietary treatments (*p* > 0.05).

**TABLE 10:** Intestinal morphometry of laying Japanese quails fed increasing dietary levels of Pracaxi oil.

Gut segments	Inclusion levels (%)					SEM^a^	*p*
	0	0.045	0.090	0.180	0.360		
Villus height, μm							
Duodenum	808.040	767.400	864.240	808.780	859.780	25.656	0.8017
Jejunum	489.470	519.490	496.820	441.360	492.360	21.332	0.8911
Ileum	360.850	471.940	359.170	340.580	406.520	36.531	0.8767
Villus width, μm							
Duodenum	228.64	248.56	227.89	254.53	216.49	6.250	0.2697
Jejunum	75.537	75.850	72.600	72.370	68.452	2.452	0.8901
Ileum	76.830	61.320	63.150	66.246	77.240	3.728	0.6120
Crypt depth, μm							
Duodenum	187.930	192.930	180.370	175.640	150.560	4.510	0.0059
Jejunum	190.110	189.290	175.730	157.720	183.780	4.715	0.1343
Ileum	176.370	184.450	164.740	151.760	176.880	5.700	0.4144
Crypt diameter, μm							
Duodenum	62.450	58.320	67.760	59.080	59.052	1.462	0.2988
Jejunum	63.946	57.813	62.250	57.077	56.323	1.457	0.3488
Ileum	51.096	48.080	53.365	51.556	50.865	1.617	0.9512
Villus:Crypt, μm/μm							
Duodenum	4.330	3.949	4.770	4.510	5.800	0.246	0.1068
Jejunum	2.630	2.980	2.726	2.836	2.890	0.131	0.9433
Ileum	2.623	2.580	2.410	2.243	2.425	0.190	0.9793
Villus width:height ratio							
Duodenum	0.290	0.333	0.260	0.306	0.250	0.014	0.3832
Jejunum	0.157	0.150	0.153	0.166	0.140	0.006	0.8543
Ileum	0.166	0.130	0.180	0.196	0.195	0.009	0.2143
Polynomial regressions							
Crypt depth		*p*	Effect	Equations			*R* ^2^
		0.0008	Linear	*y* = −112.289*x* + 192.759			0.5833

^a^ SEM = standard error of the mean. Values are presented as means of seven replicates, with one bird sampled per experimental unit. Ten well‐oriented villi and their associated crypts were measured for each intestinal segment. Linear and quadratic effects of dietary Pracaxi oil inclusion levels were evaluated by polynomial regression. Statistical significance was declared at *p* < 0.05.

## 4 Discussion

These findings indicate that, although the inclusion of Pracaxi oil did not significantly affect performance, egg quality, or biochemical parameters, it improved crude protein and mineral matter metabolizability and reduced duodenal crypt depth. Overall, these findings suggest that Pracaxi oil may influence nutrient utilization and intestinal morphology in laying Japanese quails under the conditions evaluated in the present study; however, intestinal functionality was not directly assessed.

Previous studies, such as those by Kalakuntla et al. (2017), Tavakkoli et al. (2021), and Jafari et al. (2021), have demonstrated the beneficial effects of dietary oils and supplements on intestinal health, immune response, and poultry performance, particularly with respect to fatty acid profiles and antioxidant capacity. Although these studies did not specifically evaluate Pracaxi oil, they provide a basis for understanding the potential of vegetable oils as functional feed additives in poultry nutrition.

The fatty acid profile of Pracaxi oil, as revealed in the present study, is predominantly composed of oleic, linoleic, arachidic, and behenic acids, which together account for more than 90% of its total fatty acid content. Smaller amounts of lauric (0.17%), myristic (0.09%), palmitic (1.49%), stearic (3.45%), and linolenic (1.39%) acids were also identified. These findings are consistent with previous reports highlighting the bioactive composition of Pracaxi oil, including its high antioxidant activity and substantial content of phenolic compounds (Eberhart et al. 2023). Previous toxicological evaluation demonstrated no changes in body weight, organ weight, or clinical signs after subchronic exposure; however, mild histological alterations were observed at higher doses, indicating that long‐term safety still requires further investigation (Eberhart et al. 2023).

Regarding feed efficiency, although no significant effects were observed on egg production or feed intake, increasing levels of Pracaxi oil were associated with an increase in feed conversion ratio, indicating poorer feed efficiency. This response may be attributed to the high proportion of behenic acid (23.01%), which is known for its low intestinal absorption (Yang et al. 2001). While functional oils generally enhance nutrient utilization and feed efficiency (Pourabedin and Zhao 2015; Shehata et al. 2022), the partial inhibition of pancreatic lipase by behenic acid may explain the observed decline in feed conversion (Arishima et al. 2009). The improvements observed in nutrient metabolizability were not accompanied by measurable benefits in productive performance. This apparent discrepancy may be related to the fact that the birds received nutritionally adequate diets formulated to meet their requirements for maintenance and egg production. Under such conditions, improvements in the utilization of specific nutrients may not necessarily result in detectable increases in egg production, egg weight, or egg mass. Moreover, the linear increase in feed conversion per dozen eggs indicates that the physiological responses observed for nutrient metabolizability did not translate into improved productive efficiency under the conditions of the present study.

Protein metabolizability showed a significant positive linear response to increasing dietary Pracaxi oil inclusion. Within the evaluated inclusion levels, higher concentrations of Pracaxi oil were associated with improved protein metabolizability. One possible explanation is the high oleic acid content of Pracaxi oil, as oleic acid has been reported to stimulate the release of cholecystokinin (CCK), which enhances pancreatic enzyme secretion and may improve protein digestion and nutrient utilization (Damgaard et al. 2013).

Phytotherapeutic compounds, such as functional oils and essential oils, are widely recognized for improving crude protein metabolizability, mainly by promoting intestinal health, an effect attributed to the presence of bioactive components (Mahgoub et al. 2019; Abdel‐Wareth and Lohakare 2020). In this context, Su et al. (2021) observed a significant improvement in digestibility coefficients with the use of essential oils, associated with increased production and activity of digestive enzymes, as well as enhanced expression of sodium–glucose cotransporters (SGLT) and glucose transporters, particularly in the jejunum and ileum, thereby favoring nutrient transport and absorption.

Additionally, Gholami‐Ahangran et al. (2022) reported that the inclusion of essential oils is associated with increased secretion of pepsin and hydrochloric acid, which enhances protein digestion and consequently improves mineral utilization. These effects reinforce the role of essential oils in optimizing digestive and absorptive processes.

The bioactive compounds present in Pracaxi oil, particularly polyphenols, have the potential to modulate the intestinal microbiota, strengthen immune function, and regulate cytokine production, all of which may contribute to improved intestinal functionality (Santos et al. 2020; Klein 2021). Although these mechanisms were not directly evaluated in the present study, they may have contributed to the positive linear responses observed for crude protein and mineral matter metabolizability with increasing dietary Pracaxi oil inclusion.

The increase in mineral matter metabolizability is particularly relevant, as minerals play essential metabolic, structural, energetic, and immunological roles (Goff 2018). The linear increase in mineral matter metabolizability observed in the present study suggests that Pracaxi oil may contribute to improved mineral utilization in laying quails. Although studies involving Pracaxi oil remain limited, research on Amazonian oils with similar compositions, such as açaí oil, which is rich in oleic and linoleic acids, has not reported adverse effects on egg quality in commercial laying hens (Fortuoso et al. 2020). These findings suggest that the fatty acid profile and bioactive compounds of Pracaxi oil did not compromise egg quality in the present study, corroborating results observed with oils of similar characteristics.

Regarding egg quality, the results showed no significant impact on the evaluated parameters at the tested levels of Pracaxi oil inclusion, confirming that the bioactive compounds in the oil did not interfere with egg production or quality. Fatty acid analysis of quail egg yolks revealed the presence of several fatty acids found in Pracaxi oil. However, only linolenic acid, a minor component (1.39%), exhibited a significant quadratic response, with the fitted regression equation predicting the maximum deposition at a dietary Pracaxi oil inclusion level of 0.170%. This effect may be attributed to the antioxidant properties of Pracaxi oil, likely associated with its flavonoid content and various polyunsaturated fatty acids (PUFAs), which may help mitigate lipid peroxidation in egg yolks (Cimrin et al. 2019).

With respect to intestinal morphology, the inclusion of Pracaxi oil resulted in reduced crypt depth in the duodenum, which may indicate alterations in intestinal epithelial turnover. Crypts, located between the base of the villus and the muscularis mucosae, are responsible for enterocyte proliferation and epithelial renewal and play a central role in maintaining mucosal integrity (Gava et al. 2015).

Similar results were reported by Cheng et al. (2022), who observed reduced crypt depth associated with a lower rate of cellular turnover, allowing nutrients to be redirected toward other metabolic functions. In the present study, the reduction in duodenal crypt depth observed with Pracaxi oil inclusion may reflect changes in epithelial morphology similar to those previously described, although reduced crypt depth has been associated with lower epithelial turnover and improved nutrient utilization in previous studies (Cheng et al. 2022; Ibrahim 2011).

Increased crypt depth may indicate an adaptive response to stimuli or irritation of the intestinal mucosa, aimed at preserving villus height (Lemos et al. 2013; Wu et al. 2018). Therefore, Pracaxi oil may have influenced epithelial morphology in the duodenal crypts, as evidenced by the reduction in crypt depth with increasing dietary oil levels in quails.

Overall, dietary Pracaxi oil produced limited and mixed responses under the conditions evaluated. Although crude protein and mineral matter metabolizability increased and duodenal crypt depth decreased, these responses were not accompanied by improvements in productive performance. Further studies are required to clarify the biological mechanisms underlying these responses and to determine the practical applicability of Pracaxi oil in laying Japanese quail nutrition.

## 5 Conclusion

Dietary Pracaxi oil supplementation had no effect on egg production, egg quality, serum biochemical parameters, or organ biometry of laying Japanese quails under the conditions of the present study. However, feed conversion ratio per dozen eggs increased linearly with increasing dietary Pracaxi oil inclusion. In contrast, crude protein and mineral matter metabolizability increased linearly, while duodenal crypt depth decreased, indicating changes in intestinal morphology that may be associated with nutrient utilization, although intestinal functionality was not directly evaluated in the present study. The quadratic regression model estimated a maximum predicted yolk α‐linolenic acid (C18:3 ω3) concentration of 0.182% at a dietary Pracaxi oil inclusion level of 0.170%. Overall, dietary Pracaxi oil produced limited and mixed responses under the conditions evaluated. Although some responses were observed in nutrient metabolizability, intestinal morphology, and yolk fatty acid composition, these effects were not accompanied by improvements in productive performance, and feed conversion per dozen eggs increased with increasing inclusion levels. Therefore, further studies evaluating a wider range of dietary inclusion levels and the biological mechanisms involved are needed before practical recommendations can be established.

## Author Contributions

B.S. Eberhart, J.K. Valentim, and C.M. Komiyama contributed to the conceptualization of the study. Data curation was performed by B.S. Eberhart, M.F.C. Burbarelli, and F.C. Serpa. Formal analysis was conducted by B.S. Eberhart. Funding acquisition was carried out by R.G. Garcia and C.M. Komiyama. Investigation involved B.S. Eberhart, M.F.C. Burbarelli, V.A.R.C.H. Heiss, F.C. Serpa, G.A. Felix, and F.B. Bacha. Methodology was developed by J.K. Valentim, R.G. Garcia, and C.M. Komiyama. Project administration was performed by B.S. Eberhart. Resources were provided by P.H. Braz, C.A.L. Cardoso, E.R.S. Gandra, R.G. Garcia, and C.M. Komiyama. Supervision was conducted by C.M. Komiyama and C.A.L. Cardoso. Validation was performed by J.K. Valentim and C.M. Komiyama. Visualization was prepared by M.F.C. Burbarelli and V.A.R.C.H. Heiss. The original draft was written by B.S. Eberhart and J.K. Valentim. Writing – review and editing were performed by A.A. Almeida.

## Funding

This work was supported by Coordenação de Aperfeiçoamento de Pessoal de Nível Superior (001) and Conselho Nacional de Desenvolvimento Científico e Tecnológico (150188/2022‐6).

## Conflicts of Interest

The authors declare no conflicts of interest.

## Data Availability

The data supporting the findings of this study are available upon request from the corresponding author.
